# Follicular Regulatory T Cells Can Access the Germinal Center Independently of CXCR5

**DOI:** 10.1016/j.celrep.2019.12.076

**Published:** 2020-01-21

**Authors:** Ine Vanderleyden, Sigrid C. Fra-Bido, Silvia Innocentin, Marisa Stebegg, Hanneke Okkenhaug, Nicola Evans-Bailey, Wim Pierson, Alice E. Denton, Michelle A. Linterman

**Affiliations:** 1 Laboratory of Lymphocyte Signalling and Development, Babraham Institute, Cambridge CB22 3AT, UK; 2 Imaging Facility, Babraham Institute, Cambridge CB22 3AT, UK; 3 Biological Services Unit, Babraham Research Campus, Cambridge CB22 3AT, UK

**Keywords:** follicular regulatory T cells, germinal center response, CXCR5

## Abstract

The germinal center (GC) response is critical for generating high-affinity humoral immunity and immunological memory, which forms the basis of successful immunization. Control of the GC response is thought to require follicular regulatory T (Tfr) cells, a subset of suppressive Foxp3^+^ regulatory T cells located within GCs. Relatively little is known about the exact role of Tfr cells within the GC and how they exert their suppressive function. A unique feature of Tfr cells is their reported CXCR5-dependent localization to the GC. Here, we show that the lack of CXCR5 on Foxp3^+^ regulatory T cells results in a reduced frequency, but not an absence, of GC-localized Tfr cells. This reduction in Tfr cells is not sufficient to alter the magnitude or output of the GC response. This demonstrates that additional, CXCR5-independent mechanisms facilitate Treg cell homing to the GC.

## Highlights

• CXCR5-deficient Tfr cells can migrate into the B cell follicle and the germinal center
• Absence of CXCR5 on Treg cells reduces the number of Tfr cells by half
• Halving Tfr cell numbers does not affect germinal center B cell or Tfh cell frequency

## Graphical Abstract

**Figure:**
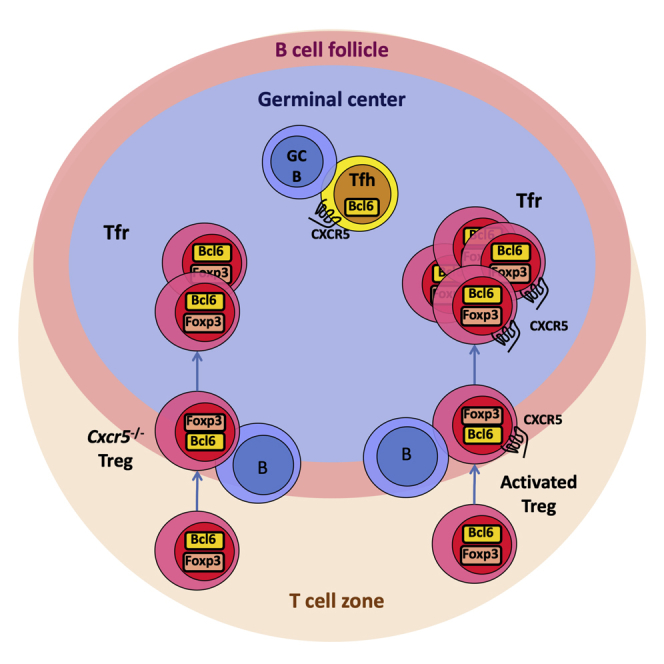

## Teaser

Vanderleyden et al. show that CXCR5-deficient Treg cells can migrate into the B cell follicle. In the absence of CXCR5, fewer Tfr cells participate in the germinal center reaction, but the reduction in Tfr cell number does not affect the magnitude of the germinal center response.

## Introduction

Follicular regulatory T (Tfr) cells are a distinct subset of Foxp3^+^ regulatory T (Treg) cells that are located within the germinal center (GC), in which they are thought to suppress the magnitude and output of the GC response (Botta et al., 2017, Chung et al., 2011, Fu et al., 2018, Kawamoto et al., 2014, Linterman et al., 2011, Sage et al., 2016, Vanderleyden et al., 2014, Wollenberg et al., 2011, Wu et al., 2016). Tfr cells phenotypically resemble T follicular helper (Tfh) cells in many aspects, including the expression of programmed cell death protein 1 (PD-1), C-X-C chemokine receptor type 5 (CXCR5), B cell lymphoma 6 (Bcl6), Slam-associated protein (SAP), and inducible costimulator (ICOS) (Chung et al., 2011, Linterman et al., 2011, Wollenberg et al., 2011). However, Tfr cells do not express the B cell helper molecules interleukin (IL)-21, IL-4, and CD40L but instead express Treg signature molecules such as GITR, CTLA-4, and Foxp3 (Chung et al., 2011, Linterman et al., 2011, Sage et al., 2014, Wing et al., 2014, Wollenberg et al., 2011). Gene expression analysis shows that Tfr cells have a distinct transcriptional profile that is more similar to that of Treg cells than to that of Tfh cells or other T helper cell subsets (Linterman et al., 2011, Wing et al., 2017). Furthermore, Tfr cells have suppressive function and are therefore considered a subset of Treg cells that are thought to regulate the GC response (Linterman et al., 2011, Stebegg et al., 2018, Wing et al., 2017).

Given the central role of the GC response in generating highly effective humoral immune responses and immunological memory, it is of considerable biological interest to understand how Tfr cells function within this response (Vanderleyden et al., 2014). Although the field has grown exponentially in recent years, relatively little is known about the exact role of Tfr cells within the GC and the mechanisms through which they exert their suppressive function. Although initial studies agreed that Tfr cells can limit the size of the GC response, they lacked a system to genetically remove Tfr cells while leaving other Tfh and Treg cells intact (Chung et al., 2011, Linterman et al., 2011, Wollenberg et al., 2011). Therefore, we set out to develop a mouse model that specifically lacks Tfr cells without affecting Tfh cells or other Treg cell subsets. A unique feature of Tfr cells is their position within the GC, which discriminates them from other Treg cell subsets, and this localization was reported to depend on CXCR5-driven chemotaxis toward the GC (Chung et al., 2011, Wollenberg et al., 2011). Therefore, genetic removal of *Cxcr5* in Foxp3^+^ Treg cells is a logical approach for generating a mouse model that specifically lacks Tfr cells and would enable the study of the GC response in the absence of Tfr cells.

To this end, we developed three mouse strains that lack CXCR5 either in all Foxp3^+^ Treg cells or in all T cells: *Cxcr5*^*fl/fl*^*Foxp3*^*cre-yfp*^ mice, *Cxcr5*^*fl/fl*^*Foxp3*^*cre-ERT2*^ mice, and *Cxcr5*^*fl/fl*^*Cd4*^*cre/+*^ mice (Bradford et al., 2017, Fontenot et al., 2005, Rubtsov et al., 2010). To our surprise, despite successful depletion of CXCR5 on Treg cells, Tfr cells were still present in the GC after immunization. However, loss of CXCR5 reduced the number of Tfr cells within the GC, indicating that it is partially required for Treg cell localization to the GC but that it is not necessary. Altogether, this demonstrates that CXCR5-independent mechanisms exist that allow Treg cell localization to the GC.

## Results

### *Cxcr5*^*fl/fl*^*Foxp3*^*cre*^ Mice Have Foxp3^+^ Cells within the GC

**Figure 1:**
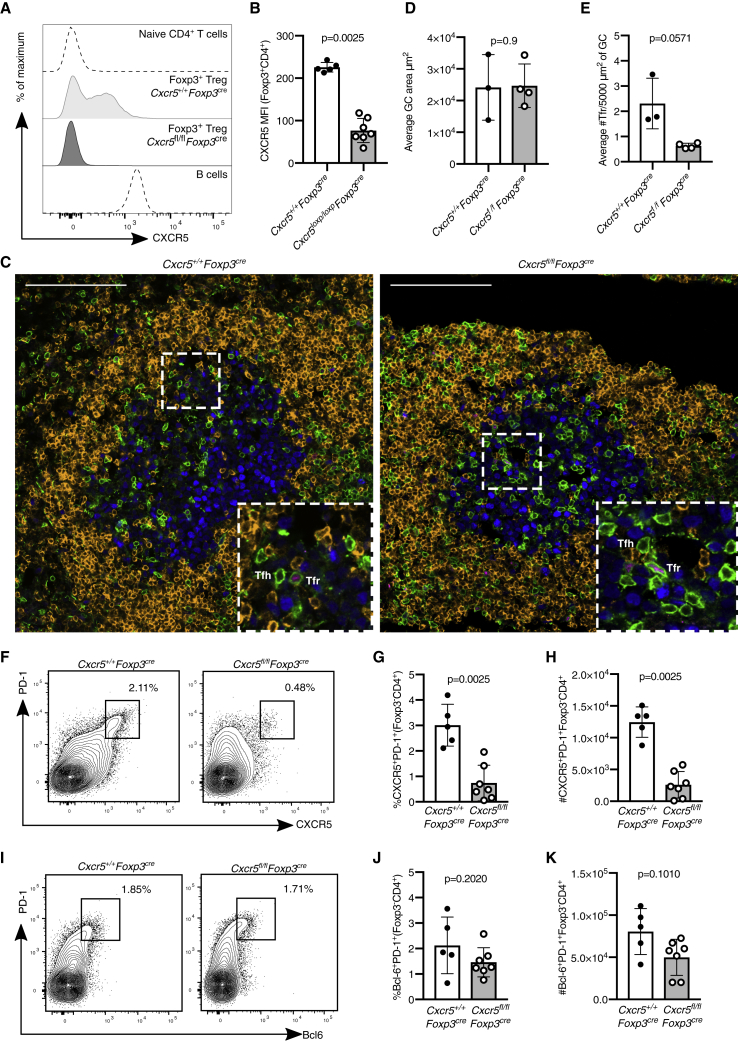
Tfr Cells Are Present at Reduced Numbers in *Cxcr5*^*fl/fl*^*Foxp3*^*cre-yfp*^ Mice Mice were immunized with NP-KLH/alum i.p., and the GC response was analyzed 14 days after immunization. (A) Histogram of CXCR5 expression in Foxp3^+^CD4^+^ Treg cells, naive T cells as a CXCR5-negative control population, and wild-type B cells as a CXCR5-positive population. (B) CXCR5 mean fluorescence intensity (MFI; geometric mean) in Foxp3^+^CD4^+^ Treg cells from mice of the indicated genotypes. (C) Analysis of Tfr and Tfh cells 14 days after influenza A virus (HKx31) infection in *Cxcr5*^*fl/fl*^*Foxp3*^*cre*^ mice and *Cxcr5*^+/+^*Foxp3*^*cre*^ controls. Representative confocal images of splenic cryosections stained for Foxp3 (magenta), Ki67 (blue), CD3 (green), and IgD (orange); Foxp3^+^ cells are indicated by arrows. Scale bar, 40 μm. (D) Average GC size in square micrometers measured as the IgD^−^Ki67^+^ area. Each dot represents the average size of 2–6 GCs per mouse. (E) Quantification of the average number of Tfr cells per mouse, defined as CD3^+^Foxp3^+^ cells within the GC, per 5,000 μm^2^. Each dot represents the average number of Tfr cells per 5,000 μm^2^ of GC area per mouse, from 2–6 GCs. (F) Representative flow cytometry contour plots of CXCR5^+^PD-1^+^ Tfh cells from Foxp3^−^CD4^+^ cells. (G and H) Quantification of the (G) frequency and (H) absolute number of CXCR5^+^PD-1^+^ Tfh cells. (I) Representative flow cytometry contour plots of Bcl6^+^PD-1^+^ Tfh cells of Foxp3^−^CD4^+^ cells. (J and K) Quantification of the (J) percentage and (K) absolute number of Bcl6^+^PD-1^+^Foxp3^−^CD4^+^ Tfh cells. Each symbol represents one mouse, the horizontal bars represent mean values, and the error bars show the SD. The p values were determined using a Mann-Whitney U test. Data represent two independent experiments.

To remove *Cxcr5* from Foxp3^+^ Treg cells, we crossed *Cxcr5*^*fl/fl*^ mice, in which exon 2 of *Cxcr5* was flanked by two *loxP* sites, with *Foxp3*^*cre-yfp*^ mice (Bradford et al., 2017, Fontenot et al., 2005). *Cxcr5*^*fl/fl*^*Foxp3*^*cre*^ mice were immunized intraperitoneally (i.p.) with 4-hydroxy-3-nitrophenylacetyl (NP)-keyhole limpet hemocyanin (KLH)/alum, and the GC response in the spleen was analyzed 14 days after immunization. CXCR5 was deleted from Foxp3^+^ Treg cells in *Cxcr5*^*fl/fl*^*Foxp3*^*cre*^ mice (Figures 1A and 1B). To determine whether Tfr cells were present in the GC in the absence of CXCR5, we enumerated the GC area and CD3^+^Foxp3^+^ Treg cells present within the GC (IgD^−^Ki67^+^) by confocal imaging (Figure 1C). There was no difference in GC area between *Cxcr5*^*fl/fl*^*Foxp3*^*cre*^ and control mice (Figure 1D). Surprisingly, Foxp3^+^ Tfr cells could still be identified in cryosections of the spleen of *Cxcr5*^*fl/fl*^*Foxp3*^*cre*^ mice, although their numbers were reduced by half compared with *Cxcr5*^+/+^*Foxp3*^*cre*^ control animals (Figures 1E, S1A, and S1B). Although the reduction of Tfr cells in *Cxcr5*^*fl/fl*^*Foxp3*^*cre*^ mice was modest, we hypothesized that this may result in impaired suppression of Tfh cells and thus an increase in the number of Tfh cells. However, fewer CXCR5^+^PD-1^+^ Tfh cells were identified in *Cxcr5*^*fl/fl*^*Foxp3*^*cre*^ mice compared with controls (Figures 1F–1H). When Tfh cells were identified using a CXCR5-independent gating strategy based on coexpression of Bcl6 and PD-1, we observed normal frequencies and absolute numbers of Tfh cells in *Cxcr5*^*fl/fl*^*Foxp3*^*cre*^ mice (Figures 1I–1K). This indicates that there may be deletion of CXCR5 from Foxp3-negative cells in the *Cxcr5*^*fl/fl*^*Foxp3*^*cre*^ mice. Consistent with this, we observed that some B cells from these mice lacked CXCR5 (Figures S1C and S1D). Both B cells and Tfh cells use CXCR5 for migration to the GC; therefore, non-specific deletion of *Cxcr5* in *Cxcr5*^*fl/fl*^*Foxp3*^*cre*^ mice limits the ability to draw conclusions about the impact of the reduced frequency of Tfr cells on the GC response. Consequently, an alternative approach for deleting *Cxcr5* specifically from Foxp3^+^ Treg cells was required to determine the impact that loss of CXCR5 from Treg cells has on the GC response.

### Specific Deletion of CXCR5 in *Cxcr5*^*fl/fl*^*Foxp3*^*cre-ERT2*^ Mice Does Not Prohibit Tfr Cell Localization to the GC

**Figure 2:**
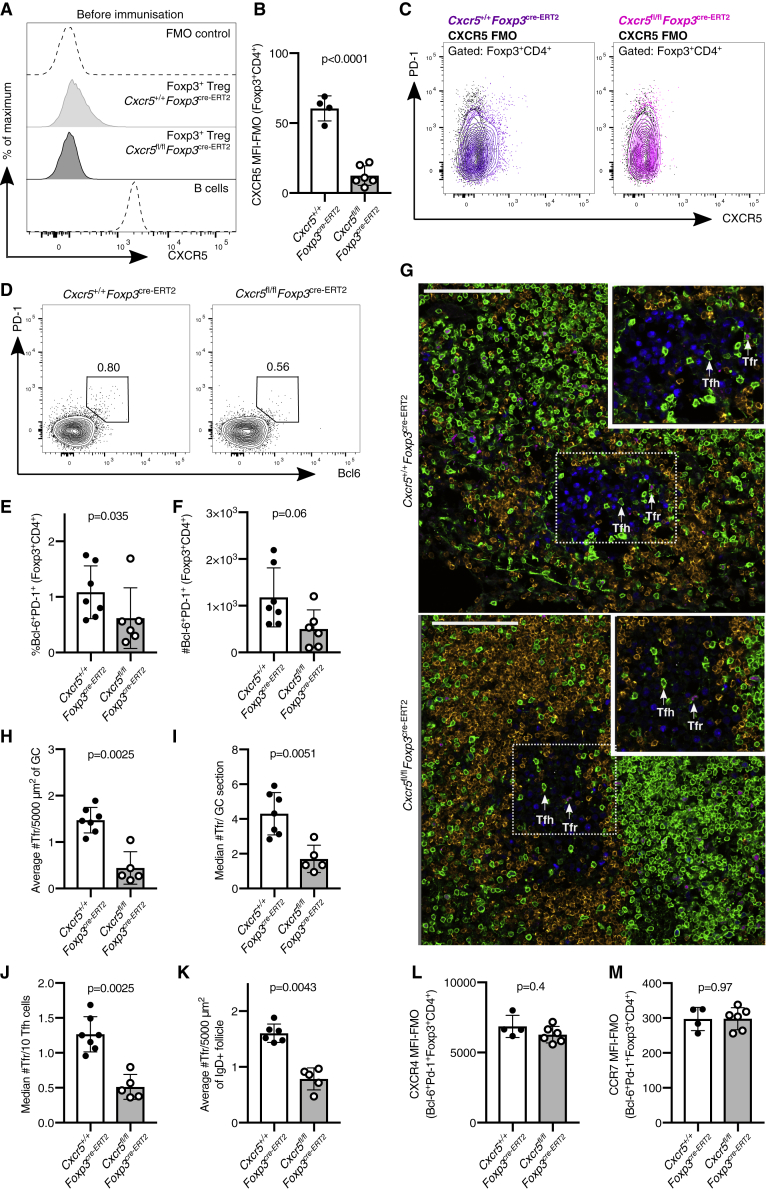
Fewer Tfr Cells Are Present in the GC of *Cxcr5*^*fl/fl*^*Foxp3*^*cre-ERT2*^ Mice (A–C) Histogram (A), quantification (B). and dot plots (C) of CXCR5 expression on Foxp3^+^CD4^+^ Treg cells in mesenteric lymph nodes three weeks after initiating the tamoxifen diet, before immunization, in *Cxcr5*^*fl/fl*^*Foxp3*^*cre-ERT2*^ and *Cxcr5*^+/+^*Foxp3*^*cre-ERT2*^ mice. A fluorescence minus one (FMO) control serves as a negative control, and B220^+^ B cells serve as a CXCR5-positive population. (D–M) Mice were immunized with NP-KLH/alum s.c., and the GC response was analyzed in draining lymph nodes 14 days after immunization. (D) Representative flow cytometry contour plots of PD-1^+^Bcl6^+^ cells within Foxp3^+^CD4^+^ cells (Tfr cells). (E and F) Quantification of the (E) percentage and (F) absolute number of Bcl6^+^PD-1^+^ Tfr cells. (G) Cryosections from iLNs were stained for Foxp3 (magenta), Ki67 (blue), CD3 (green), and IgD (orange). Scale bar, 100 μm. Representative confocal image of the GC, with Tfr cells and Tfh cells indicated by the arrows. (H) Quantification of the median number of Tfr cells, defined as CD3^+^Foxp3^+^, per 5,000 μm^2^ of GC area. (I) Quantification of confocal images of the median number of CD3^+^Foxp3^+^ Tfr cells per GC per mouse. (J) Quantification of the median number of Tfr cells per 10 Tfh cells, defined as Foxp3^−^CD3^+^. (K) Quantification of the median number of Treg cells, defined as CD3^+^Foxp3^+^, per 5,000 μm^2^ of IgD^+^ B cell follicle area. (L and M) CXCR4 MFI (L) and CCR7 MFI (M) (subtracting FMO control) on Bcl6^+^PD-1^+^Foxp3^+^CD4^+^ Tfr cells from mice of the indicated genotypes. Each symbol represents one mouse, the horizontal bars represent mean values, and the error bars show the SD. The p values were determined using a Mann-Whitney U test. For the quantification of confocal images, 4–10 GCs or B cell follicles were imaged per mouse. Data represent two or more independent experiments.

In an attempt to limit CXCR5 deletion specifically to Treg cells, we crossed *Cxcr5*^*fl/fl*^ mice with *Foxp3*^*cre-ERT2*^ mice, which have a cre recombinase linked to a mutated estrogen receptor ligand binding domain (ERT2) inserted in the 3′ untranslated region of the *Foxp3* gene, allowing inducible deletion of the floxed allele upon tamoxifen administration (Rubtsov et al., 2010). To induce cre recombinase activity in Treg cells, mice received a tamoxifen-containing diet for 5 weeks to induce deletion of CXCR5 from Treg cells. *Cxcr5* was successfully deleted from Foxp3^+^ cells of *Cxcr5*^*fl/fl*^*Foxp3*^*cre-ERT2*^ mice after three weeks of tamoxifen treatment (Figures 2A–2C), without loss of CXCR5 from B cells in these animals (Figure S1E). Mice treated with tamoxifen for three weeks were then immunized with NP-KLH/alum subcutaneously (s.c.) in the flank, followed by analysis of the inguinal lymph node (iLN). Despite the loss of CXCR5 from Treg cells, Bcl6^+^PD-1^+^ Tfr cells could still be identified in *Cxcr5*^*fl/fl*^*Foxp3*^*cre-ERT2*^ mice, with ∼40% the frequency of control mice 14 days after immunization (Figures 2D–2F). A reduction in Bcl6^+^PD-1^+^ Tfr cells in *Cxcr5*^*fl/fl*^*Foxp3*^*cre-ERT2*^ mice was also observed seven days after immunization (Figures S2A–S2E). However, flow cytometric analysis cannot rule out that Treg cells have a Tfr cell phenotype form but are unable to localize to the GC. Therefore, the presence of Foxp3^+^ cells within the GC was analyzed in cryosections from iLN by confocal imaging (Figures 2G and S3). Tfr cells could clearly be identified within the GC of *Cxcr5*^*fl/fl*^*Foxp3*^*cre-ERT2*^ mice, and quantification of the number of Tfr cells normalized to total GC area, per GC, or to the number of Tfh cells showed a 3-fold reduction in the number of Tfr cells within the GC but not absence of these cells (Figures 2H–2J). This finding is consistent with our results from the *Cxcr5*^*fl/fl*^*Foxp3*^*cre*^ mice (Figure 1) but is discordant with previous reports, which showed that CXCR5-deficient Treg cells did not localize to the GC after adoptive transfer into T cell-deficient hosts (Chung et al., 2011). In addition, a two-fold reduction in the number of Foxp3^+^CD3^+^ cells in the immunoglobulin (Ig) D^+^ B cell follicle was observed in *Cxcr5*^*fl/fl*^*Foxp3*^*cre-ERT2*^ mice, suggesting that CXCR5 is important for localization to the B cell follicle, although the effect size is less than for Treg localization to the GC (Figure 2K). Collectively, these data demonstrate that the deletion of CXCR5 from Treg cells is not sufficient to impair their access to the GC, suggesting that additional mechanisms to guide these cells to the GC may exist.

Analysis of publicly available RNA sequencing data of CD25^−^ GC-localized Tfr cells (Wing et al., 2017) demonstrated that the most highly expressed chemokine receptor on GC-Tfr cells is *Cxcr4* (Figure S4). CXCR4 is highly expressed on the surface of Tfr cells in both *Cxcr5*^*fl/fl*^*Foxp3*^*cre-ERT2*^ and control mice 14 days after immunization (Figure 2L), suggesting that this receptor could facilitate Tfr cell homing to the CXCL12-rich area of the GC when CXCR5 is lacking (Denton and Linterman, 2017). The chemokine receptor that is most lowly expressed on Tfr cells, compared with their Treg precursors, is CCR7 (Figure S4), a receptor whose downregulation is essential for Tfh cell localization to the GC (Haynes et al., 2007). CCR7 expression is not altered by the lack of CXCR5 in *Cxcr5*^*fl/fl*^*Foxp3*^*cre-ERT2*^ mice (Figure 2M). Altogether, this demonstrates that loss of CXCR5 does not alter the expression of two highly differentially expressed chemokine receptors on Tfr cells, which may facilitate their localization to the GC in the absence of CXCR5.

### The Output and the Size of the GC Is Unaltered in *Cxcr5*^*fl/fl*^*Foxp3*^*cre-ERT2*^ Mice

**Figure 3:**
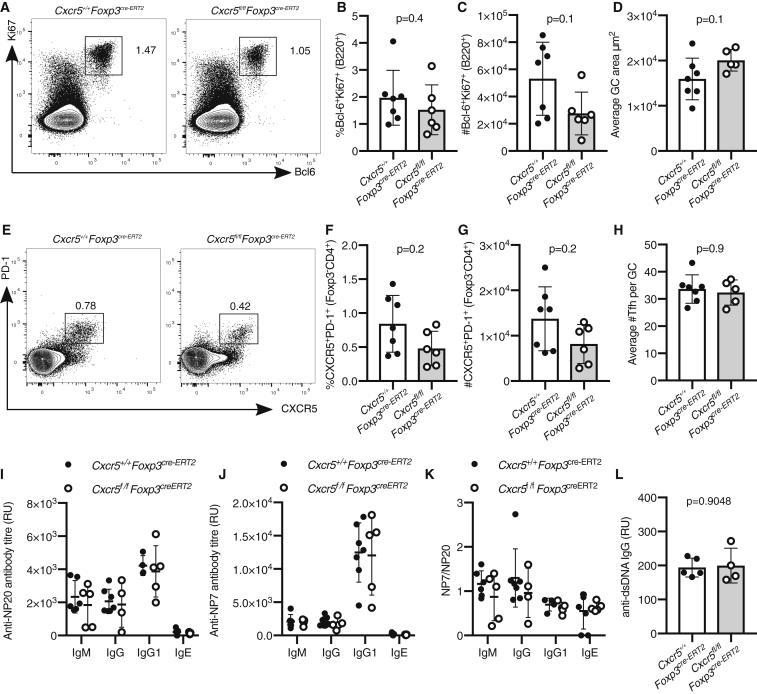
Normal Magnitude and Output of the GC Response in *Cxcr5*^*fl/fl*^*Foxp3*^*cre-ERT2*^ Mice Analysis of the GC response 14 days after s.c. immunization with NP-KLH/alum. (A) Flow cytometry contour plots of Bcl6^+^Ki67^+^B220^+^ GC B cells. (B and C) Quantification of the (B) frequency and (C) absolute number of Bcl6^+^Ki67^+^ B cells. (D) Quantification of confocal images of the average GC area per mouse of the indicated genotypes. (E) Flow cytometry contour plots of Tfh cells, gated as CXCR5^+^PD-1^+^ of Foxp3^−^CD4^+^ cells. (F and G) Quantification of the (F) percentage and (G) number of CXCR5^+^ Tfh cells. (H) Quantification of confocal images of the average number of CD3^+^Foxp3^−^ Tfh cells per GC area per mouse of the indicated genotypes. (I) Levels of anti-NP7 antibodies of the indicated isotypes in the sera. (J) Levels of anti-NP20 antibodies of the indicated isotypes in the sera. (K) Ratio of NP20/NP7 of the indicated isotypes in the sera. (L) Serum levels of IgG specific for dsDNA. Each symbol represents one mouse, the horizontal bars represent mean values, and the error bars show the SD. The p values were determined using a Mann-Whitney U test. For the quantification of confocal images, 4–10 GCs were imaged per mouse. Data represent two independent experiments.

To test whether the reduced presence of Tfr cells influenced the size and output of the GC in *Cxcr5*^*fl/fl*^*Foxp3*^*cre-ERT2*^ mice, the numbers of GC B cells and Tfh cells in *Cxcr5*^*fl/fl*^*Foxp3*^*cre-ERT2*^ mice were quantified. At both seven and fourteen days after immunization, the numbers of Ki67^+^Bcl6^+^ GC B cells were comparable in *Cxcr5*^*fl/fl*^*Foxp3*^*cre-ERT2*^ and control mice (Figures 3A–3C and S2F–S2H) as were the average GC areas measured by confocal imaging (Figure 3D). Likewise, the frequency and number of PD-1^+^CXCR5^+^Foxp3^−^ Tfh cells were measured by flow cytometry (Figures 3E–3G and S2I–S2K), and the number of CD3^+^Foxp3^−^ Tfh cells per GC was quantified by confocal imaging (Figure 3H); neither were altered in *Cxcr5*^*fl/fl*^*Foxp3*^*cre-ERT2*^ mice compared with controls. Notably, we did not observe deletion of CXCR5 from conventional CD4^+^ cells (Figure 3E), indicating Treg-specific removal. To test whether a reduction in the number of Tfr cells changed antibody production or affinity maturation, serum levels of NP-specific antibodies were assessed (Figures 3I–3K). Analysis of anti-NP antibodies of different isotypes showed that both the titer and the quality of the humoral immune response were comparable between *Cxcr5*^*fl/fl*^*Foxp3*^*cre-ERT2*^ and control mice (Figures 3I–3K). Tfr cells have previously been implicated in the prevention of the outgrowth of autoreactive B cell clones within the GC (Botta et al., 2017, Fu et al., 2018). However, *Cxcr5*^*fl/fl*^*Foxp3*^*cre-ERT2*^ mice did not have elevated levels of IgG specific for double-stranded DNA (dsDNA) in the serum (Figure 3J), suggesting a reduction in Tfr cells does cause a break of GC tolerance that results in autoantibody formation after immunization. Altogether, this demonstrates that loss of ∼60% of the GC-Tfr cell pool and a 50% reduction of Foxp3^+^ cells in the B cell follicle are not sufficient to alter the magnitude or output of the GC response.

### Tfh and Tfr Cells Are Able to Form in *Cxcr5*^*fl/fl*^*Cd4*^*cre/+*^ Mice

**Figure 4:**
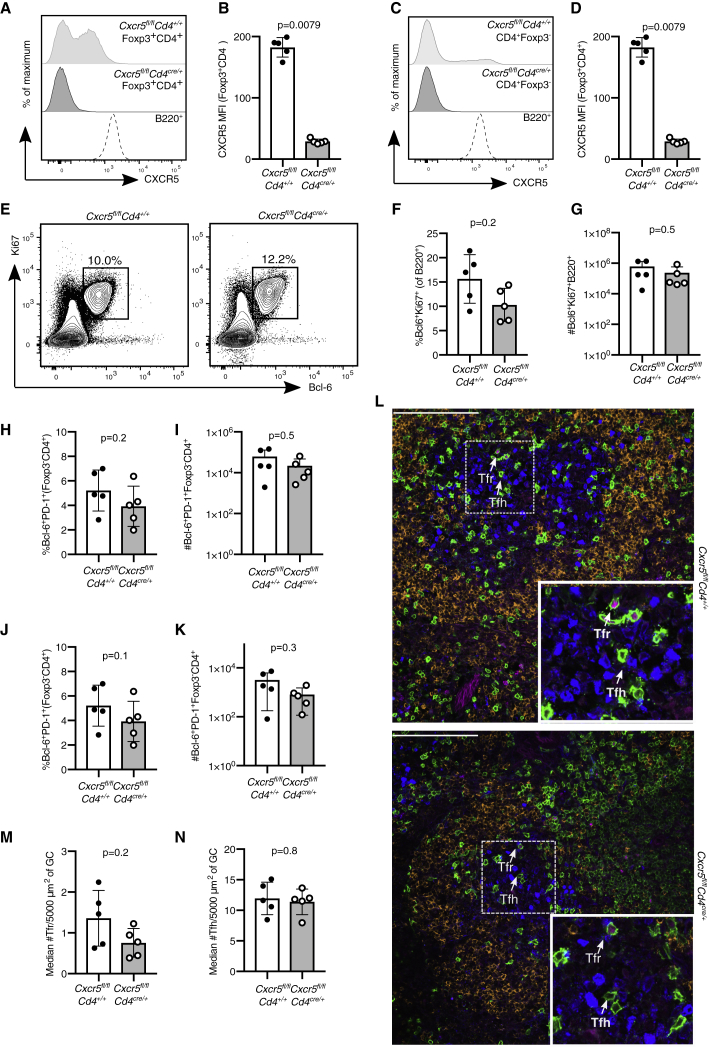
*Cxcr5*^*fl/fl*^*Cd4*^*cre/+*^ Mice Have an Intact GC Response after Influenza Infection Analysis of the GC response in *Cxcr5*^*fl/fl*^*Cd4*^*cre/+*^ mice 14 days after influenza A virus (HKx31) infection. (A) Representative histogram of CXCR5 expression in Foxp3^+^CD4^+^ Treg cells and B220^+^ B cells. (B) Quantification of the MFI (geometric mean) of CXCR5 in Foxp3^+^CD4^+^ Treg cells. (C) Representative histogram of CXCR5 expression in Foxp3^−^CD4^+^ T cells and B220^+^ B cells. (D) Quantification of the MFI (geometric mean) of CXCR5 in Foxp3^−^CD4^+^ T cells. (E) Flow cytometry contour plots of GC B cells, gated as Bcl6^+^Ki67^+^ cells of B220^+^ cells. (F and G) Quantification of the (F) frequency and (G) absolute number of Bcl6^+^Ki67^+^ B cells. (H and I) Quantification of the (H) percentage and (I) number of Bcl6^+^PD-1^+^Foxp3^−^CD4^+^ Tfh cells. (J and K) Quantification of the (J) percentage and (K) number of Bcl6^+^PD-1^+^Foxp3^+^CD4^+^ Tfr cells. (L) Cryosections were stained for Foxp3 (magenta), Ki67 (blue), CD3 (green), and IgD (orange). Scale bar, 100 μm. Representative confocal image of the GC, with Tfr cells and Tfh cells indicated by the arrows. (M) Quantification of the median number of Tfr cells, defined as CD3^+^Foxp3^+^ cells, per 5,000 μm^2^. (N) Quantification of the median number of Tfh cells, defined as CD3^+^Foxp3^−^ cells, per 5,000 μm^2^. Each symbol represents one mouse, the horizontal bars represent mean values, and the error bars show the SD. The p values were determined using a Mann-Whitney U test. For the quantification of confocal images, 4–10 GCs were imaged per mouse. Data represent two independent experiments.

The observation that Tfr cells are able to enter the GC independently of CXCR5 in both *Cxcr5*^*fl/fl*^*Foxp3*^*cre-ERT2*^ and *Cxcr5*^*fl/fl*^*Foxp3*^*cre*^ mice was unexpected, because CXCR5 had previously been reported to be essential for Treg cell migration to the GC (Chung et al., 2011, Wollenberg et al., 2011). Therefore, we wished to corroborate our observations in a third, independent mouse model in which all T cells lack CXCR5: *Cxcr5*^*fl/fl*^*Cd4*^*cre/+*^ mice. Fourteen days after influenza A virus infection, we confirmed deletion of *Cxcr5* from Foxp3^+^ Treg cells (Figures 4A and 4B) and Foxp3^−^CD4^+^ T cells (Figures 4C and 4D). Despite the loss of CXCR5 from all CD4 T cells, the frequency of Ki67^+^Bcl6^+^ GC B cells was comparable to that in control mice with intact CXCR5 (Figures 4E–4G). Analysis of Tfh and Tfr cells based on coexpression of PD-1 and Bcl6 further showed no differences between *Cxcr5*^*fl/fl*^*Cd4*^*cre/+*^ and control mice (Figures 4H–4K). Confocal image analysis confirmed the presence of both Tfh and Tfr cells within the GC of *Cxcr5*^*fl/fl*^*Cd4*^*cre/+*^ mice (Figures 4L–4N), consistent with previous reports that show that CXCR5 is not essential for GC access by Foxp3^−^CD4^+^ T cells (Moriyama et al., 2014). Altogether, these data demonstrate that lack of CXCR5 is insufficient to impair Treg cell access to the GC, suggesting that redundant mechanisms are involved in Treg cell migration to the GC.

## Discussion

Tfr cells are a specialized subset of Treg cells that access the GC, in which they are thought to exert suppressive functions. The localization of Tfr cells to the GC is thought to depend on CXCR5 migration to the CXCL13-rich B cell follicle. Here, we have used three independent mouse models that lack CXCR5 expression on Foxp3^+^ cells, all of which show the presence of Tfr cells within the GC. This reduction in Tfr cells in the GC did not alter the size of the GC response or the antibody response upon immunization, suggesting that a reduced number of Tfr cells does not recapitulate the phenotype of mice that lack Tfr cells or that have CXCR5^+^ Treg cells deleted early after immunization (Botta et al., 2017, Clement et al., 2019, Wu et al., 2016). These data suggest that CXCR5 is not the only mechanism by which Tfr cells can access the GC and that redundant mechanisms facilitate the localization of Foxp3^+^ cells to the B cell follicle.

These were unexpected results, because adoptively transferred CXCR5-deficient Treg cells into T cell-deficient mice did not migrate to the GC after immunization (Chung et al., 2011, Wollenberg et al., 2011). In this study, we used intact mouse models, rather than cell isolation and subsequent transfer, and this approach has the advantage of requiring less experimental manipulation. The differences between experimental approaches may explain the different phenotype observed. Nevertheless, the presence of CXCR5-deficient Tfr cells within the GC suggests that there are other ways by which Treg cells can access the B cell follicle.

For conventional CD4^+^Foxp3^−^ T cells to migrate to the GC, the concurrent upregulation of CXCR5 and downregulation of CCR7 is required to enable migration from the T cell zone that is rich in CCR7 ligands (Haynes et al., 2007). The retention of Tfh cells within the GC is regulated by sphingosine-1-phosphate receptor 2 (S1PR2), and lack of both S1PR2 and CXCR5 abrogates Tfh cell localization to the GC (Moriyama et al., 2014). S1PR2 is also highly upregulated in Tfr cells (Moriyama et al., 2014, Wing et al., 2017). Therefore, it is possible that S1PR2 is able to facilitate the localization of Treg cells within the GC in *Cxcr5*^*fl/fl*^*Foxp3*^*cre-ERT2*^ mice. Other homing receptors that are expressed on Tfr cells—such as CXCR4, whose ligand CXCL12 is expressed by the dark zone stromal cells within the GC—could be part of the redundant mechanisms involved in Tfr localization to the GC (Denton and Linterman, 2017). The multiple mechanisms by which Treg cells migrate to different locations within tissues have likely evolved to ensure that these important suppressive cells can get to where they need to be in the absence of just one migratory cue. An understanding of the mechanisms by which Treg cells can enter the GC may facilitate strategies to manipulate Tfr cells in both health and disease.

## STAR★Methods

### Key Resources Table

**Table:** 

REAGENT or RESOURCE	SOURCE	IDENTIFIER
**Antibodies**		

Anti-B220	Biolegend	RA3-6B2. Cat#103241; RRID: AB_11204069
Anti-B220	Biolegend	RA3-6B2. Cat#103246; RRID: AB_2563256
Anti-Bcl6	BD	K112-91 Cat#561522; RRID: AB_10717126
Anti-Bcl6	BD	K112-91. Cat#561525; RRID: AB_10898007
Anti-CD4	Biolegend	RM4-5. Cat#100547; RRID: AB_11125962
Anti-CD4	Biolegend	RM4-5. Cat#100528; RRID: AB_312729
Anti-CD4	ThermoFisher Scientific	GK1.5. Cat#17-0041-83; RRID: AB_469320
Anti-CD4	ThermoFisher Scientific	GK1.5;. Cat#48-0041-82; RRID: AB_464893
Anti-CD4	ThermoFisher Scientific	GK1.5;. Cat#11-0041-85; RRID: AB_464892
Anti-CD4	ThermoFisher Scientific	GK1.5. Cat#48-0042-82; RRID: AB_1107001
Anti-CD4	ThermoFisher Scientific	GK1.5. Cat#45-0042-82; RRID: AB_1107001
Anti-CD44	Biolegend	IM7. Cat#103020; RRID: AB_493683
Anti-CXCR5	Biolegend	L138D7. Cat#145506; RRID: AB_2561970
Anti-CXCR5	Biolegend	L138D7. Cat#145512; RRID: AB_2562128
Anti-Foxp3	ThermoFisher Scientific	FJK-16S. Cat#53-5773-82; RRID: AB_763537
Anti-Foxp3	ThermoFisher Scientific	FJK-16S. Cat#48-5773-82; RRID: AB_1518812
Anti-Ki67	ThermoFisher Scientific	SolA15. Cat#56-5698-82; RRID: AB_2637480
Anti-Ki67	ThermoFisher Scientific	SolA15. Cat#11-5698-82; RRID: AB_11151330
Anti-Ki67	ThermoFisher Scientific	SolA15. Cat#25-5698-82; RRID: AB_11220070
Anti-PD-1	Biolegend	RMP1-30. Cat#109104; RRID: AB_313421
Anti-PD-1	Biolegend	RMP1-30. Cat#109110; RRID: AB_572017
Anti-IgG1	Abcam	Cat#ab97240; RRID: AB_10695944
Anti-IgM	Abcam	Cat#ab97230; RRID: AB_10688258
Anti-IgG	Abcam	Cat#205719; RRID: AB_2755049
Anti IgE	BD Biosciences	Cat# 553419; RRID: AB_394850
Anti-Foxp3	eBioscience	FJK-16s. Cat # 53-5773-80; RRID: AB_469916
Anti-IgD	BioLegend	11-26c.2a. Cat# 405707; RRID: AB_893529
Anti-Ki67	eBioscience	SolA15. Cat #48-5698-82; RRID: AB_11149124
Anti-CD3e	eBioscience	eBio500A2. Cat# 14-0033-82; RRID: AB_837128
Goat anti-Hamster IgG (H+L)	Invitrogen	Cat# A-21112; RRID: AB_2535761
Anti-CCR7	ThermoFisher Scientific	4B12. Cat# 12-1971-82; RRID: AB_465905
Anti-CXCR4	Biolegend	L276F12. Cat# 146506; RRID: AB_2562783

**Bacterial and Virus Strains**		

A/HK/x31 (H3N2)	A gift from Prof. Douglas Fearon	N/A

**Chemicals, Peptides, and Recombinant Proteins**		

NP-KLH Conjugation ratio 29-33	Biosearch Technologies	cat#N-5060
Imject Alum Adjuvant	ThermoFisher Scientific	cat#77161
Brilliant Stain buffer	BD Horizon	cat#563794
Zombie aqua fixable viability dye	Biolegend	cat# 423101
NP7-BSA	Biosearch Technologies	cat#N-5050L-100
NP20-BSA	Biosearch Technologies	cat#N-5050H-100

**Critical Commercial Assays**		

eBioscience Foxp3/ Transcription Factor Fixation/Permeabilisation Staining buffer set	ThermoFisher Scientific	cat# 00-5523-00
3,3′,5,5′-Tetramethylbenzidine (TMB) substrate set	Biolegend	cat#421101

**Deposited Data**		

RNA-sequencing data from Wing et al., 2017	Wing et al., 2017	https://www.ncbi.nlm.nih.gov/bioproject/PRJDB5396; https://www.ncbi.nlm.nih.gov/bioproject/PRJDB4935

**Experimental Models: Organisms/Strains**		

Mouse: Cxcr5fl/fl	Roslin Institute	Bradford et al., 2017
Mouse: Foxp3cre-yfp	JAX	Rubtsov et al., 2008. Stock No: 016959
Mouse: Foxp3EGFP-cre-ERT2	JAX	Rubtsov et al., 2010. Stock No: 016961
Mouse: Cd4cre	Taconic Biosciences	Lee et al., 2001. Model#4196
Mouse: Cxcr5fl/fl Foxp3cre-YFP	This paper	N/A
Mouse: Cxcr5fl/fl Foxp3EGFP-cre-ERT2	This paper	N/A
Mouse: Cxcr5fl/f Cd4cre	This paper	N/A

**Software and Algorithms**		

FlowJo	Treestar	https://www.flowjo.com/
Volocity	PerkinElmer	https://www.perkinelmer.com/category/image-analysis-software
Graphpad Prism	Graphpad	https://www.graphpad.com/

**Other**		

16% global protein rodent diet	Teklad	cat#2916
CRD TAM400/CreER Tamoxifen pellets	Teklad	cat#TD.130860

### Lead contact and materials availability

Further information and request for resources and reagents should be directed to and will be fulfilled by the Lead Contact, Michelle Linterman (michelle.linterman@babraham.ac.uk). This study generated three new mouse strains by intercrossing *Cxcr5*^*fl/fl*^ mice (Bradford et al., 2017) with the following strains: *Foxp3*^*cre-yfp*^ (Rubtsov et al., 2010), *Foxp3*^*EGFP-cre-ERT2*^ (Rubtsov et al., 2010), and *Cd4*^*cre*^ (Lee et al., 2001). There are restrictions to the availability of the newly generated strains as all four orginal strains were obtained under material transfer agreement that does not include permission to redistribute these strains without an appropriate contract in place with the strain owner.

### Experimental model and subject details

#### Mice

The following mice were used in this study: *Cxcr5*^*fl/fl*^ (Bradford et al., 2017), *Foxp3*^*cre-yfp*^ (Rubtsov et al., 2010), *Foxp3*^*EGFP-cre-ERT2*^ (Rubtsov et al., 2010), *Cd4*^*cre*^ (Lee et al., 2001), *Cxcr5*^*fl/fl*^ *Foxp3*^*cre-YFP*^*, Cxcr5*^*fl/fl*^ *Foxp3*^*EGFP-cre-ERT2*^*,* and *Cxcr5*^*fl/f*^ *Cd4*^*cre*^ mice. All mice were on the C57BL/6J background and both males and females were used throughout. Mice were between three and 12 weeks old at the start of the experiment, and age- and sex-matched controls were used, unless stated otherwise. Mice were bred and maintained in the Babraham Institute Biological Support Unit. No primary pathogens or additional agents listed in the FELASA recommendations were detected during health monitoring surveys of the stock holding rooms. Ambient temperature was ∼19-21°C and relative humidity 52%. Lighting was provided on a 12-hour light: 12 hour dark cycle including 15 min ‘dawn’ and ‘dusk’ periods of subdued lighting. After weaning, mice were transferred to individually ventilated cages (GM 500: Techniplast) with 1-5 mice per cage. Mice were fed CRM (P) VP diet (Special Diet Services, cat#801722) *ad libitum* and received seeds (e.g., sunflower, millet) at the time of cage-cleaning as part of their environmental enrichment. All mouse experimentation was approved by the Babraham Institute Animal Welfare and Ethical Review Body. Animal husbandry and experimentation complied with existing European Union and United Kingdom Home Office legislation and local standards.

### Method details

#### Immunisation and Influenza Infection

NP-KLH (Conjugation ratio 29-33, Biosearch Technologies, cat#N-5060) was dissolved in PBS to 1 mg/ml and mixed with Imject Alum Adjuvant (ThermoFisher Scientific, cat#77161) in a 1:1 ratio by vortexing to a final working concentration of 0.5 mg/ml. Mice were immunized either; *s.c*. on each side of the hind flank with 100 μL per flank of NP-KLH/Alum under anesthesia using isoflurane, and inguinal LN were harvested 7 or 14 days after immunisation, or *i.p.* with 100 μL of NP-KLH/Alum and the spleen harvested 14 days after immunisation. Blood was collected after euthanasia in each experiment by cardiac puncture to determine NP-specific antibody production. For influenza infection, mice were inoculated intranasally (*i.n.*) with 10^4^ plaque-forming units of the influenza A/HK/x31 (H3N2, a gift from Prof. Douglas Fearon) virus under inhalation anesthesia using isoflurane. The mediastinal LN was harvested 14 days post infection.

#### Tamoxifen Treatment

Inducible deletion of floxed alleles mediated by the cre-recombinase linked to a human mutated estrogen ligand binding receptor (ERT2) was achieved by supplementing the food with tamoxifen. From the age of three weeks, mice received a soy-free, 16% global protein rodent diet (Teklad, cat#2916) for ten days. After ten days, mice were fed *ad libitum* up until six weeks with Tecklad CRD TAM^400/CreER^ Tamoxifen pellets (Teklad, cat#TD.130860), containing 400mg tamoxifen citrate/kg (w/v), softened in 20% (w/v) sucrose (Fisher chemicals, CAS 57-50-1) in water solution.

#### Flow cytometry

Flow cytometry was performed on a Fortessa (BD) and analyzed with FlowJo software (Treestar). A single cell suspension was prepared by pressing the LN through a 40-um cell strainer (BD cat#352340) in 2% fetal bovine serum (Sigma, cat#F9665) in PBS before antibody staining in Brilliant Stain buffer (BD Horizon, cat#563794). For optimal CXCR5 staining, buffers without the sodium azide were used as this preservative reduces CXCR5 detection on T cells. Antibodies used were as follows: B220 (RA3-6B2, Biolegend), Bcl6 (K112-91, BD), CD4 (RM4-5, Biolegend), CD4 (GK1.5, ThermoFisher Scientific), CD44 (IM7, Biolegend), CXCR5 (L138D7, Biolegend), Foxp3 (FJK-16S, ThermoFisher Scientific), Ki67 (SolA15, ThermoFisher Scientific), PD-1 (RMP1-30, Biolegend), CCR7 (4B12, BD or eBiosciences), CXCR4 (L276F12, Biolegend). Cells were fixed and permeabilised for intracellular staining using the eBioscience Foxp3/ Transcription Factor Fixation/Permeabilisation Staining buffer set (ThermoFisher Scientific, cat# 00-5523-00) according to manufacturer’s instructions. Dead cells were excluded by using the zombie aqua fixable viability dye (Biolegend, cat# 423101).

#### Immunofluorescence imaging

Preparation of frozen LN samples and immunofluorescence staining was performed as described previously (Vanderleyden and Linterman, 2017). LN were fixed in periodate-lysine-paraformaldehyde (1% PFA, 0.075 M L-lysine, 0.37 M sodium phosphate (pH 7.4), and 0.01 M NaIO4) for 4 hours at 4°C, incubated in sucrose 30% overnight at 4°C and embedded in optimal cutting temperature medium (FisherScientific, cat#23-730-571). Tissue sections were cut at 10 μm using a cryostat (Leica) and air-dried overnight. Prior to staining, LN sections were rehydrated in 0.5% (v/v) Tween 20 in PBS and blocked with PBS+ 2% (w/v) BSA (bovine serum albumin) + 10% (v/v) normal goat serum (NGS) and permeabilised in PBS + 2% (v/v) Triton X for 30 min at RT. Images were acquired with a Zeiss 780 microscope using 20x and 40x objectives. Image analysis was performed using Volocity (PerkinElmer). Antibodies used were as follows: rat anti-mouse/rat Foxp3 (FJK16S, ThermoFisher Scientific), rat anti-mouse Ki67 (SolA15, ThermoFisher Scientific), rat anti-mouse IgD (11-26c.2a, Biolegend), hamster anti-mouse CD3ε purified (500A2, ThermoFisher Scientific) and goat anti-hamster IgG (LifeTechnologies, cat#A-21112). Image analysis was performed using Volocity (PerkinElmer), or CellProfiler (Lamprecht et al., 2007).

#### ELISA

For the NP-specific ELISA, Nunc Maxisorb 96-well plates (ThermoFisher Scientific, cat# 44-2404-21) were coated with NP7-BSA (Biosearch Technologies, cat#N-5050L-100) at 2.5 μg/ml or NP20-BSA (Biosearch Technologies, cat#N-5050H-100) at 10 μg/ml, and incubated overnight at 4°C. To determine serum levels of IgM, IgG or IgE specific for dsDNA, Nunc Maxisorb 96-well plates were coated with 100 μL poly-l-lysine solution at 20 μg/ml (Sigma, cat # P4832) overnight at 4°C. Serum samples were serially diluted, and Horseradish Peroxidase (HRP) conjugated goat anti-mouse IgG1 (Abcam, cat#ab97240), IgM (Abcam, cat#ab97230), IgG (Abcam, cat#205719) or biotinylated rat anti-mouse IgE (BD Biosciences, cat# 553419) and HRP-conjugated Streptavidin (Southern Biotech cat#7100-05) were added. Plates were developed using the 3,3′,5,5′-Tetramethylbenzidine (TMB) substrate set (Biolegend, cat#421101). Plates were read at 450nm using a PHERAstar FS plate reader (BMG Labtech).

#### RNA sequencing analysis

RNA sequencing analysis was performed using the SeqMonk software package (Babraham Institute, https://www.bioinformatics.babraham.ac.uk/projects/seqmonk/) after trimming (Trim Galore v0.4.2) and alignment of reads to the reference mouse genome GRCm38 using HISAT2. Reads were quantitated over exons and library size was standardized to 1 million reads, and then read counts were log2 transformed.

### Quantification and statistical analysis

#### Statistical analysis

Statistical tests were chosen in advance as part of the experimental design. Sample sizes were determined in advance based on the availability of age-matched experimental mice and controls. Unpaired comparisons were performed using the Mann-Whitney U test (GraphPad Prism, version 8, GraphPad LLC). All data points were analyzed and outliers were not removed unless there were technical errors. Data are presented as the mean, with error bars indicating the standard deviation and with single data points shown. p < 0.05 was used as a threshold for statistical significance.

### Data and code availability

This study did not generate datasets.

## Supplemental Information

Document S1. Figures S1–S4

Document S2. Article plus Supplemental Information
