# Supplementary material for: Follicular Regulatory T Cells Can Access the Germinal Center Independently of CXCR5
Source: Cell Rep. 2020 Jan 21;30(3):611–619.e4. doi: 10.1016/j.celrep.2019.12.076 (PMC6988108; doi:10.1016/j.celrep.2019.12.076)
Supplement: Document S1. Figures S1–S4 [file mmc1.pdf]

Cell Reports, Volume *30*

Supplemental Information

Follicular Regulatory T Cells Can Access the Germinal Center Independently of CXCR5

Ine Vanderleyden, Sigrid C. Fra-Bido, Silvia Innocentin, Marisa Stebegg, Hanneke Okkenhaug, Nicola Evans-Bailey, Wim Pierson, Alice E. Denton, and Michelle A. Linterman

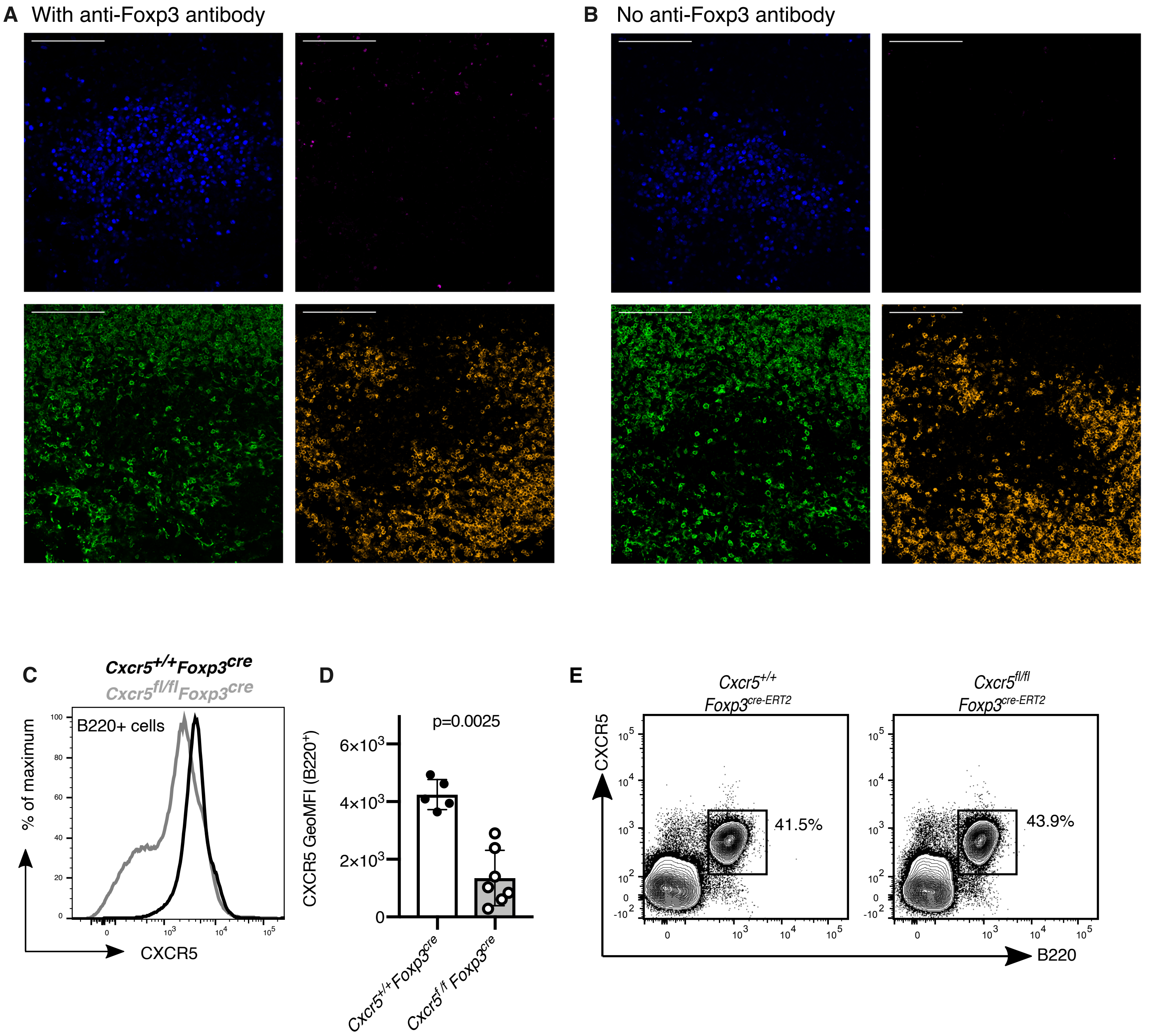

Figure S1, confocal staining controls and CXCR5 staining on B cells, related to Figures 1 and 2: (A-B) Confocal staining control for Foxp3. **(A)** Confocal image of the GC stained for Foxp3 (magenta), Ki67 (blue), CD3 (green) and IgD (orange), or **(B)** the same stain in the absence of Foxp3 staining to enable non-specific detection to be identified. Scale bar = 100 µm. **(C-D)** Non-specific deletion of CXCR5 in *Cxcr5$^{fl/fl}$Foxp3^{cre}* mice. Representative flow cytometry histogram **(C)** and quantification of **(D)** CXCR5 expression on B220^{+} B cells on splenic B cells from mice of the indicated genotype. **(E)** *Cxcr5$^{fl/fl}$Foxp3$^{cre\text{-}ERT2}$* mice show specific deletion of CXCR5 in Foxp3^{+} Treg cells. Analysis of CXCR5 expression on B cells 14 days after *s.c.* immunisation with NP-KLH/Alum. **(E)** Representative flow cytometry contour plots of B220^{+} B cells from single cells from mice of the indicated genotypes. Each symbol represents one mouse, error bars show standard deviation, and the horizontal bars represent the median values. P values were determined using a Mann-Whitney U test. Data represent two independent experiments.

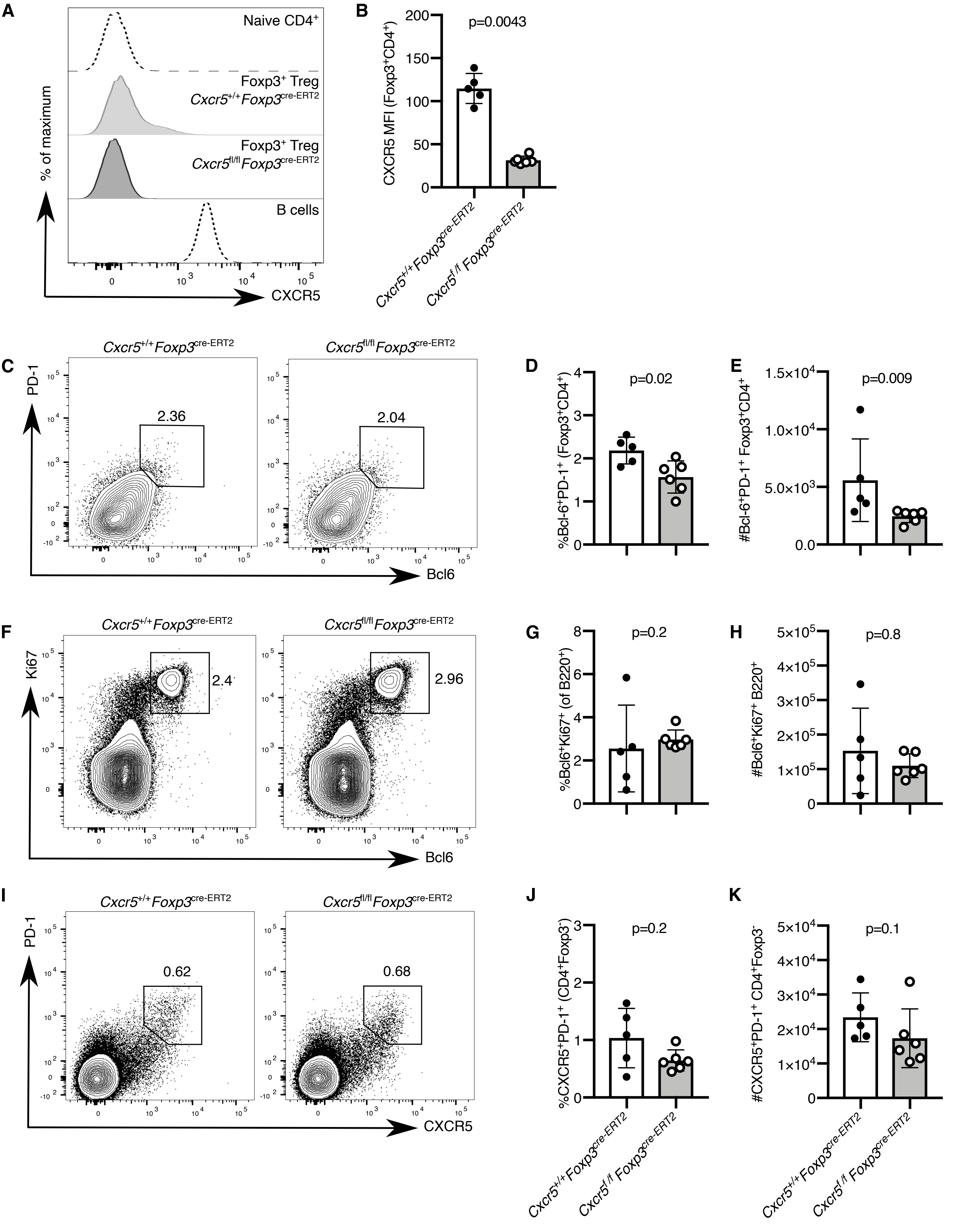

Figure S2, germinal centre responses in *Cxcr5$^{fl/fl}$Foxp3$^{cre-ERT2}$* mice seven days after immunization, related to Figures 2 and 3: Fewer Tfr cells, but normal Tfh and GC B cell numbers in *Cxcr5$^{fl/fl}$Foxp3$^{cre-ERT2}$* mice seven days after immunization. Analysis of the GC response in the inguinal lymph node seven days after *s.c.* immunisation with NP-KLH/Alum. Histogram (A) and quantification (B) of CXCR5 expression on Foxp3$^+$CD4$^+$ Treg cells in inguinal lymph nodes in *Cxcr5$^{fl/fl}$Foxp3$^{cre-ERT2}$* and *Cxcr5$^{+/+}$Foxp3$^{cre-ERT2}$* mice who have been on the tamoxifen diet for five weeks and immunised with NP-KLH/Alum. Naïve T cells (CD44^{low}CD4$^+$Foxp3$^-$) serve as a negative control and B220$^+$ B cells as a CXCR5-positive population. (C) Representative flow cytometry plots of PD-1$^+$Bcl6$^+$ cells within Foxp3$^+$CD4$^+$ cells (Tfr cells). Quantification of the (D) percentage and (E) absolute number of Bcl6$^+$PD-1$^+$ Tfr cells. (F) Flow cytometry contour plots of Bcl6$^+$Ki67$^+$B220$^+$ GC B cells. Quantification of (G) the frequency and (H) absolute number of Bcl6$^+$Ki67$^+$ B cells. (I) Flow cytometry plots of Tfh cells, gated as CXCR5$^+$PD-1$^+$ of Foxp3$^-$CD4$^+$ cells. Quantification of (J) the percentage and (K) number of CXCR5$^+$ Tfh cells. Each symbol represents one mouse, and the horizontal bars represent the median values, error bars represent the standard deviation. P values were determined using a Mann-Whitney U test. Data representative of two independent experiments with 5-6 mice per group.

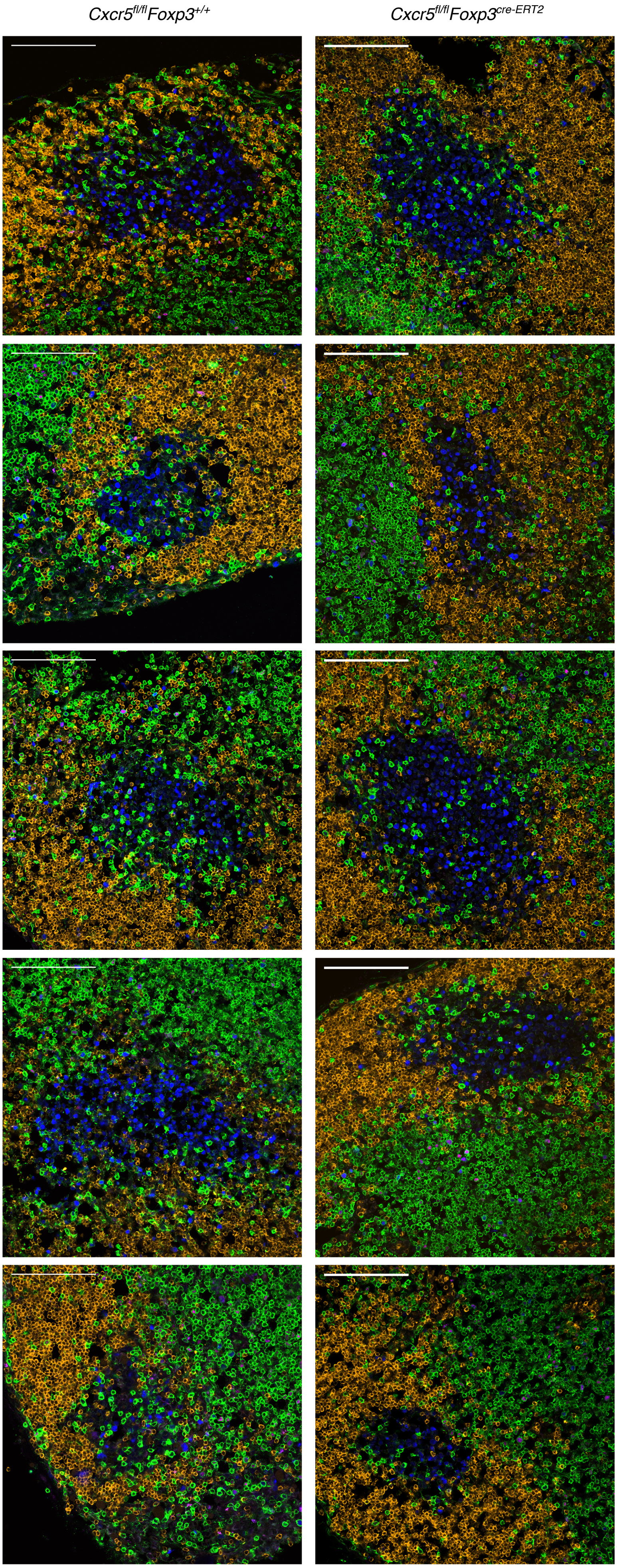

Figure S3, fewer Tfr cells are present in the germinal centre of *Cxcr5^fl/fl^Foxp3^cre-ERT2^* mice, related to Figures 2 and 3: Representative confocal images of the GC in the inguinal LN 14 days after NP-KLH/Alum s.c. immunisation. Cryosections were stained for Foxp3 (magenta), Ki67 (blue), CD3 (green) and IgD (orange). Scale bar = 100 µm.

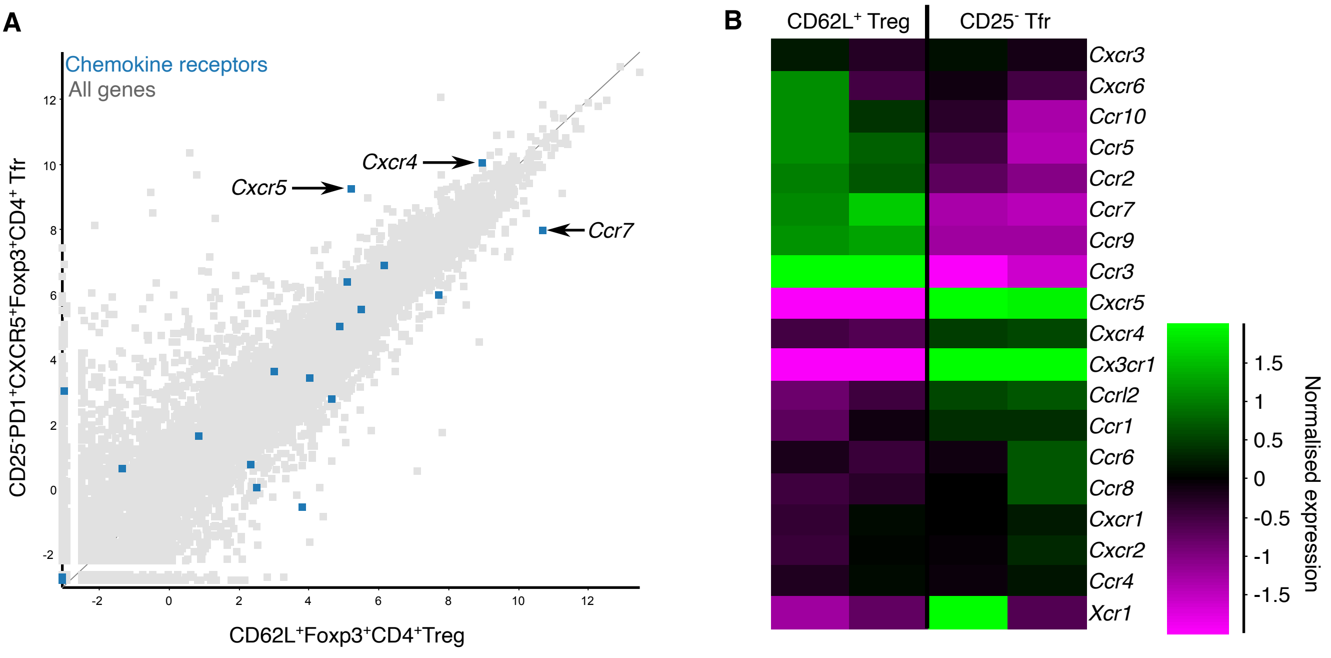

Figure S4, CXCR4 and CCR7 are differentially expressed on Tfr cells compared to naïve Treg precursors, related to Figures 2 and 3: RNAseq analysis of $CD25^-CXCR5^+PD\text{-}1^+Foxp3^+CD4^+$ Tfr cells and $CD62L^+Foxp3^+CD4^+$ naïve Treg cells. (A) Scatter plot of all mRNAs, those encoding chemokine receptors are shown in blue and all other genes shown in grey. (B) Heat map of relative chemokine receptor mRNA expression in Tfr and naïve Treg cells. Data are reanalysed from Wing et al (Wing et al., 2017).
